# An observational study in an urban Ugandan clinic comparing virological outcomes of patients switched from first-line antiretroviral regimens to second-line regimens containing ritonavir-boosted atazanavir or ritonavir-boosted lopinavir

**DOI:** 10.1186/s12879-019-3907-5

**Published:** 2019-03-25

**Authors:** Eva Agnes Odongpiny Laker, Maria Sarah Nabaggala, Arvind Kaimal, Damalie Nalwanga, Barbara Castelnuovo, Abdu Musubire, Agnes Kiragga, Mohammed Lamorde, Rosalind Parkes- Ratanshi

**Affiliations:** 1 Infectious Diseases Institute, Makerere University College of Health Sciences, P.O. Box 22418, Kampala, Uganda; 2 Institute of Public Health, University of Cambridge, Cambridge, UK

**Keywords:** Second-line antiretroviral, First-line failure, Atazanavir, Lopinavir

## Abstract

**Background:**

The World Health Organisation approved boosted atazanavir as a preferred second line protease inhibitor in 2010. This is as an alternative to the current boosted lopinavir. Atazanavir has a lower genetic barrier than lopinavir. We compared the virological outcomes of patients during the roll out of routine viral load monitoring, who had switched to boosted second- line regimens of either atazanavir or lopinavir.

**Methods:**

This was a cross-sectional study involving adult patients at the Infectious Diseases Institute Kampala, Uganda started on a standard WHO recommended second-line regimen containing either boosted atazanavir or boosted lopinavir between 1 Dec 2014 and 31 July 2015.. Mantel -Haenszel chi square was used to test for the statistical significance of the odds of being suppressed (VL < 400 copies/ml) when on boosted atazanavir compared to boosted lopinavir after stratifying by duration on antiretroviral therapy (ART). Multivariate logistic regression analysis used to determine if the type of boosted protease inhibitor (bPI) was associated with virological outcome.

**Results:**

Ninety (90) % on ATV/r and 83% on LPV/r had a VL less than 1000 copies/ml. The odds of being suppressed using the same viral load cut-off while on boosted atazanavir compared to boosted lopinavir was not statistically significant after stratifying for duration on ART (*p* = 0.09). In a multivariate analysis the type of bPI used was not a predictor of virological outcome (*p* = 0.60).

**Conclusions:**

Patients using the WHO recommended second-line of boosted atazanavir have comparable virological suppression to those on boosted lopinavir.

## Background

The Joint United Nations Programme on HIV and AIDS (UNAIDS) 2016 report estimates that 17 million people living with HIV are accessing antiretroviral therapy (ART), 60.6% (10.3 million) of these are living in sub-Saharan Africa [1]. Employing a public health approach to HIV treatment with a simplified and standardized package of care has allowed a large number of patients to access lifesaving ART in highly under-resourced settings [2]. This public health approach recommends first-line regimens that are replaced by second-line regimens when treatment failure to the first-line regimen occurs.

Prior to 2013, treatment failure was determined using clinical criteria (e.g. new or recurrent World Health Organisation stage 4 opportunistic infections) or immunological criteria (dropping CD4 count or persistent < 100 cells/ml) or a detectable viral load (VL) greater than 5000 copies/ml. Since 2013, routine viral load monitoring has been recommended for monitoring response to ART. While the exact number of patients on second-line therapy in resource limited settings is not known, the World Health Organisation (WHO) estimates that 6% of all individuals receiving first-line therapy in sub-Saharan Africa need to switch to second-line regimens in any given year [3]. However, a meta-analysis by Yoann Madec et al. estimated that only 3% of the patients on ART in resource limited settings (RLS) receive second-line ART [4].

The recommended first-line regimen by WHO for a public health approach consists of two nucleos(t)ides reverse transcriptase inhibitors (NRTIs, preferred is tenofovir disproxil fumarate) and a non-nucleoside reverse transcriptase inhibitor (NNRTI, preferred is efavirenz 600 mg). The second-line regimen comprises of two nucleosides reverse transcriptase inhibitors (NRTIs) plus a boosted protease inhibitor (bPI) (preferred is boosted lopinavir or boosted atazanavir) [5–7]. Due to the lack of resistance testing, patients who fail first-line therapy are at risk of multiple mutations to their nucleos(t)ide reverse transcriptase inhibitors (NRTIs) and yet these are recycled in the second-line [5, 8–11]. It is known that bPIs have a high genetic barrier to resistance, therefore it is recommended that they be used alongside NRTIs to enhance efficacy of the regimen [12–14].

Guidelines initially recommended boosted lopinavir as the preferred protease inhibitor, but in 2010 the WHO revision of guidelines for ART for HIV in adults and adolescents added boosted atazanavir alongside boosted lopinavir as preferred bPIs for second-line therapy [14–16]. Whilst first-line boosted atazanavir and boosted lopinavir based regimens have been compared and proven to be equally efficacious in patients starting first-line, there are no randomized controlled trials or large observational studies that have compared the efficacy of the two drug regimens ATV/r and LPV/r in resource limited settings that follow the WHO policy with probable late diagnosis of first-line ART failure [17–19]. Further evidence for the recommendations for these drugs are based mainly on studies in resource rich settings with access to resistance testing to determine the most efficacious second-line regimens [18].

In 2011, Uganda adopted the WHO recommendation to use boosted atazanavir as a preferred alternative to boosted lopinavir [20]. The reasons for the preferential use are; the one tablet once daily dosing of boosted atazanavir and its better lipid and gastrointestinal side-effect profile. Prior to December 2014, VL testing was not performed for routine ART monitoring. In 2014, routine VL monitoring for all patients was rolled out in Uganda and this presented an opportunity to determine the outcomes of patients switched to boosted atazanavir and boosted lopinavir based regimens. We undertook a cross-sectional study of all people receiving standard second-line treatment during the roll out of routine VL monitoring between 1 Dec 2014 and 31 July 2015 at the Infectious Diseases Institute. This study aimed to describe the virological outcomes of patients who were on boosted second-line regimen of either atazanavir or lopinavir at that time.

## Methods

The Infectious Diseases Institute (IDI) in Kampala, Uganda is a center for specialist management of HIV with over 8000 patients in care, and 1200 of these were on second-line therapy. Patient medical records are maintained via an electronic system and use of all routinely collected clinic data is approved for analysis and reporting by the Makerere University Faculty of Medicine, Research and Ethics committee (approval number: 120–2009) and Uganda National Council for Science and Technology (approval number: 45683) hence the need for consent was waived by an IRB. All abstracted data was de-identified and stored on a password protected computer to protect maintain patients’ anonymity. We identified patients who were initiated on a second-line therapy of boosted atazanavir and boosted lopinavir between 01/Jan/2010 and 01/Dec/2014. We included patients aged ≥18 years, who were active (attended clinic at least once in the previous 3 months), had undergone VL testing and who had been on a standard first-line therapy comprising of two NRTIS and either nevirapine (NVP) or efavirenz (EFV) and switched to a standard second-line of two NRTIs (one new and the other recycled) and either boosted atazanavir or boosted lopinavir. These drugs are generic preparations, from WHO accredited manufacturers. All reviewed patients must have attended clinic at least once in the previous 3 months (not lost to follow up) and must have been on second-line therapy for at least 6 months. Patients who missed their routine visits in the study period were excluded as well as those who were enrolled in an ongoing observational cohort and were being followed up using non-routine VLs.

From December 2014, routine VLs were carried out at the Ministry of Health Central Public Health Laboratory (CPHL) using (Roche CAP-CTM and Abbott M2000sp/M2000rt with cut-offs of 20 copies/μL and 75 copies/ μL respectively). Results were returned back to the facility within 2 weeks.

Baseline demographic and clinical characteristics at the time of switch to second-line were compared between patients on boosted atazanavir and boosted lopinavir Categorical variables were compared using the chi-square test. The Wilcoxon rank-sum test was used to compare non-normally distributed continuous variables. Virological suppression was defined as having a VL less than 400 copies/ml.

Mantel -Haenszel chi square was used to test for the statistical significance of the odds of being suppressed when on boosted atazanavir compared to boosted lopinavir after stratifying by duration on ART. Propensity scores were generated to account for the probability of being switched to either boosted atazanavir or boosted lopinavir and the variables used to predict this were type of type of bPI, gender, age, body mass index (BMI) and CD4 at switch to second-line. At the multivariable level analysis, a logistic regression was used to determine the factors associated with suppression (VL < 400 copies/ml)., Only variables with an unadjusted *P* value less than 0.2.. Variables were considered significant at the 95% level.

## Results

**Fig. 1:**
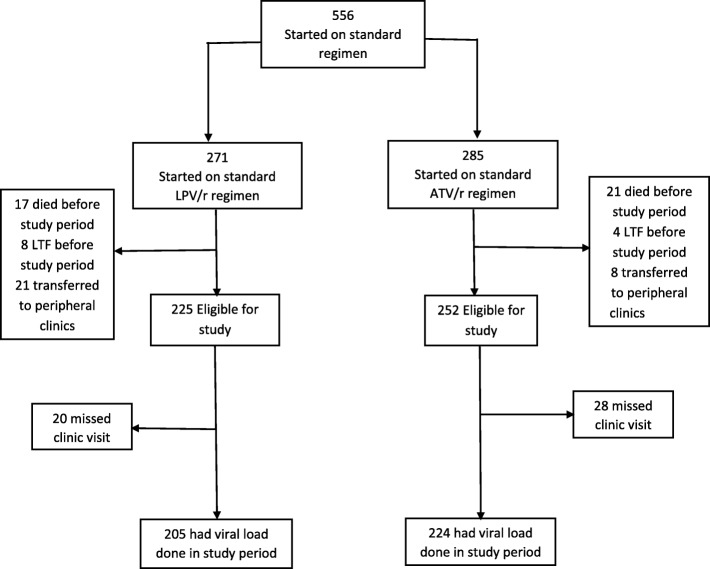
Follow up status of patients ever started on boosted atazanavir and on boosted lopinavir at time of study

A total of 285 patients had ever been started on boosted atazanavir (ATV/r) and 271 on boosted lopinavir (LPV/r) in the study period, of which 252 (88.4%) and 225 (83.0%) were still active in the clinic and 224 in the ATV/r, and 205 in the LPV/r had a VL available. The rates of loss to follow up, death and transfer outs of patients started on ATV/r and on LPV/r were similar;(33/285) 12% on ATV/r and (46/271) 17% on LPV/r (*p* = 0.315) (Fig. 1).

The majority in either group were female (ATV/r: 148 (66.1%), LPV/r: 128 (62.4%), the median age (IQR) was 37 (30–42) and 36 years (31–42) respectively for ATV/r and LPV/r. Most patients at time of switch had a normal BMI, low CD4 below 200 cells/mm^3^ and had been on nevirapine with no difference between the groups (Table 1). The median log VL in both groups at time of switch was high 4.5 (3.8–4.9) and 4.5 (3.2–5.1) respectively for ATV/r and LPV/r and not statistically different. Patients on ATV/r had been on a first-line treatment for a longer duration with a median of 56 months (28–80) as compared to 46 months (25–67) for patients on LPV/r (*p* = 0.025). The median duration (IQR) on second-line by time of VL was also shorter for patients on (ATV/r)19 months (21–25) versus 40 months on LPV/r (32–52) (*p* < 0.001)). TDF/3TC was the NRTI combination used in the majority, mostly in the group on ATV/r (85.3%) compared to 154 (75.1%) on LPV/r (*P* < 0.05) .

**Table 1:** Patient characteristics at time of switch to second-line anti-retroviral regimen

Characteristic	ATV/r	LPV/r	*P* value
Eligible	252	225	
Included, n (%)	224 (88.8)	205 (91.1)	0.663
Sex, n (%)			
Female	148 (66.1)	128 (62.4)	0.223
Male	76 (33.9)	77 (37.6)	
Age (years), median (IQR)	37 (30–42)	36 (31–42)	0.792
Grouped BMI, Kg/m^2^, n (%)^a^			
Under weight	15 (6.9)	21 (11.4)	0.305
Normal	137 (63.4)	111 (60.0)	
Over weight	64 (29.6)	53 (28.7)	
Missing	8	20	
First-line drug, n (%)			
NVP	144 (64.3)	132 (64.4)	0.982
EFV	80 (35.7)	73 (35.6)	
CD4 count cell/μl, median (IQR)	118 (59–214)	102 (59–189)	0.088
Viral load Log 10 copies/ml at switch to second- line, median (IQR)	4.5 (3.8–4.9)	4.5 (3.2–5.1)	0.421
Duration (months) on first-line therapy, median (IQR)	56 (28–80)	46 (25–67)	0.025
Duration (months on second-line by time of viral load, median (IQR)	19 (21–25)	40 (32–52)	< 0.001
NRTI drugs in second line, n (%)			
3TC-TDF	191 (85.3)	154 (75.1)	0.741
3TC-AZT	29 (12.9)	48 (23.4)	
3TC-ABC	4 (1.8)	3 (1.5)	

^a^BMI was categorized as Underweight (< 18.5 Kg/m^2^), Normal (18.5–24.9 Kg/m^2^),

Over weight (≥25.0) Kg/m^2^

**Fig. 2:**
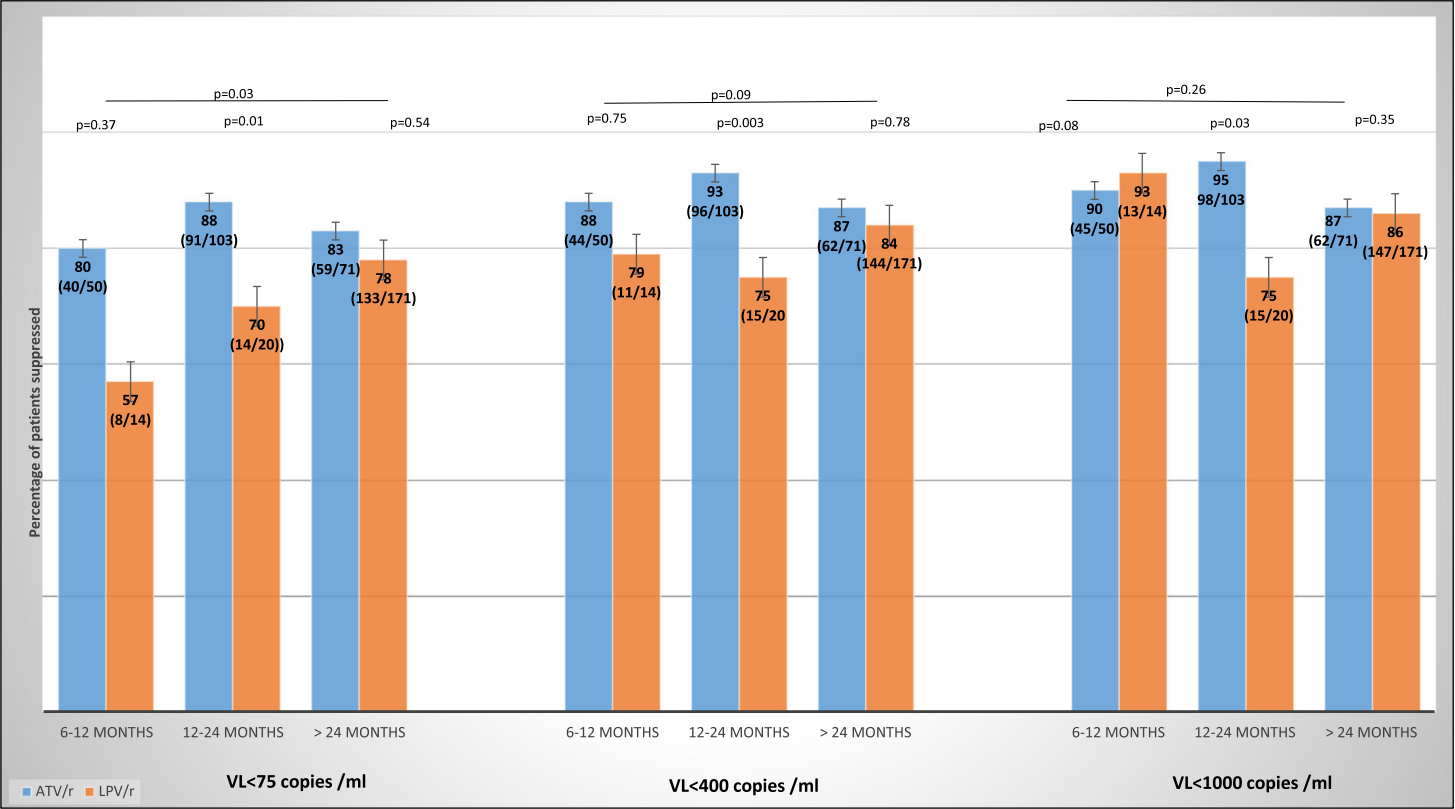
Percentage suppression with different viral load cutoffs in patients by duration on second line therapy at time of viral load (standard ATV/r versus LPV/r based second-line regimen)

90% on ATV/r and 83% on LPV/r had a VL < 400 copies /ml. However, after stratifying by time on second-line therapy this was not statistically significant (*p* = 0.09). Further sensitivity analyses using a cut-off of VL < 1000 copes/ ml resulted in 92% on ATV/r and 85% on LP/r suppressed and after stratifying by time on second-line therapy this was also not statistically significant. (*p* = 0.26). However, a cut-off of < 75 copies/ml showed that the odds of being suppressed were more favorable for ATV/r (85%) compared to LPV/r (76%) on stratifying by time on therapy (*p* = 0.03) (Fig. 2).

In a multivariate analysis controlling for the effects of sex, age at switch to second-line ART, CD4 at switch, time on first-line therapy and time from switch to second-line to VL date, the type of bPI was not associated with the outcome of virological suppression (VL < 400 copies/ml) (Table 2).

**Table 2:** Multivariate analysis by virological outcome (VL < 400 copies/ml)

Variable	Adjusted OR (95% CI)	P value
Sex		
Female	Reference	.
Male	0.63 (0.29–1.34)	0.233
Second-line regimen group		
ATV/r	Reference	.
LPV/r	0.46 (0.20–1.03)	0.060
Age at switch to second-line, per 5 year increase	0.91 (0.76–1.10)	0.332
CD4 count cell/μl, at switch to second-line	0.99 (0.99–1.00)	0.468
Time (months) on first-line therapy	1.00 (0.99–1.02)	0.701
Time (years) after switch to viral load date	1.27 (0.95–1.68)	0.103

Note: Adjusted for matching using propensity scores:

## Discussion

In this snapshot of African patients on second-line ART, we report a high proportion of patients with viral suppression on both boosted atazanavir and boosted lopinavir containing regimens. Using different VL cut-offs that is, < 1000 and < 400 copies/ml, the proportions suppressed did not differ significantly between the two groups. However, further sensitivity tests showed that the odds of being suppressed were significantly different when a cutoff of < 75 copies/ml was used (*p* = 003). However previous studies have shown that a VL < 75 copies/ml is not predictive of virological failure and therefore while patients on ATV/r are more likely to achieve a VL < 75 copies/ml this might not confer an advantage in terms of clinical outcomes [21, 22]. This trend in favor of ATV/r at lower VL cut-offs in the real-life setting could be driven by the better tolerability that would drive better adherence in those taking it compared to LPV/r. This however warrants more studies to confirm our observation [11].

Our findings are similar to findings in other studies. In Georgia a study evaluated outcomes of patients on both boosted atazanavir and boosted lopinavir containing regimens in PI-naïve patients failing first-line ART and multivariate analysis revealed that the type of bPI used was not associated with the odds of suppression [23]. Similarly, the BMS 045 study was undertaken in patients on boosted atazanavir and boosted lopinavir in resource rich settings also showed no significant differences in virological outcomes in the two groups. However, the patients in the BMS study had previously received PI regimens with some having PI mutations and resistance testing was available to guide the choice of the nucleos(t)ides used in the PI regimen [24]. Both studies used a cut-off of ≥400 copies/ml for virological failure. However, BMS also had further analyses using a cut-off of 50 copies/ml showing no difference in suppression rates between boosted atazanavir and lopinavir.

Boosted lopinavir has a higher genetic barrier than boosted atazanavir [12]. However, genetic barrier alone may not be a predictor of how a drug will perform. In the 2-LADY study, though boosted darunavir has a higher genetic barrier than boosted lopinavir, it did not meet non-inferiority criteria in patients with a VL > 100,000 copies per ml [25]. Notably our findings show that despite the lower genetic barrier, patients on boosted atazanavir are achieving high levels of suppression similar to boosted lopinavir. This could be related to its tolerability profile or ease of taking (less gastrointestinal side effects, less pills and once per day preparation) of the boosted atazanavir.

While our study is the first to the best of our knowledge to attempt to compare outcomes between the two drugs when used in a resource-limited setting, it must be emphasized that it is cross-sectional in nature. A study with longitudinal follow up of viral loads would have been less prone to survivor bias however viral load monitoring was not available for routine care till December 2014 so there was no opportunity for this. The populations differed in terms of their durations on treatment though on stratifying the virological outcomes by duration on second-line, there was still no significant differences in the odds of being suppressed when on either PI type. Patient accrual through death, loss to follow up and transfer out for stable patients were similar in both groups.

Another limitation of our study was that we were not able to control for all possible predictors of the virological outcome at the multivariate stage such as viral load at switch, opportunistic infections and adherence. Whilst a randomized study as well data from cohorts with longitudinal VL monitoring would provide the strongest evidence on non-inferiority of atazanavir, we find this observational data to be reassuring for the increasingly large numbers of patients receiving generic atazanavir in sub-Saharan Africa. In addition, since this study involved patients who had been followed up prior to ART switch without routine VLs, it is applicable to a vast number of resource-limited settings that have low coverage of VL monitoring.

## Conclusion

Boosted atazanavir regimens had comparable virological outcomes to boosted lopinavir regimens in patients failing first line therapy in settings where there has not been routine viral load monitoring. There is also a trend towards better suppression rates with boosted ATV/r when lower cut-offs (vl < 75 copies/ml) are used. This evidence supports the WHO recommendation of boosted atazanavir as a component of second-line regimens in developing countries.

## Abbreviations

ART: Antiretroviral therapy
ATV/r: Atazanavir and ritonavir combination
BMI: Body mass index
bPI: Boosted protease inhibitor
CPHL: Ministry of Health Central Public Health Laboratory
EFV: Efavirenz
IDI: Infectious Diseases Institute
LPV/r: Lopinavir and ritonavir combination
NNRTI: Non-nucleoside reverse transcriptase inhibitor
NRTI: Nucleos(t)ide reverse transcriptase inhibitor
NVP: Nevirapine
RLS: Resource limited settings
UNAIDS: Joint United Nations Programme on HIV and AIDS
VL: Viral load
WHO: World Health Organisation
